# An Active Self-Driven Piezoelectric Sensor Enabling Real-Time Respiration Monitoring

**DOI:** 10.3390/s19143241

**Published:** 2019-07-23

**Authors:** Ahmed Rasheed, Emad Iranmanesh, Weiwei Li, Yangbing Xu, Qi Zhou, Hai Ou, Kai Wang

**Affiliations:** 1 Guangdong Province Key Laboratory of Display Material and Technology, State Key Laboratory of Optoelectronic Materials and Technologies, School of Electronics and Information Technology, Sun Yat-sen University, No. 132 East Waihuan Road, Guangzhou 510006, China; 2 Sun Yat-sen University Shunde Research Institute, No. 9 Eastern Nanguo Road, Shunde District, Foshan 523800, China

**Keywords:** thin-film transistor, piezoelectric transducer, active sensor, self-driven sensor, respiration monitoring

## Abstract

In this work, we report an active respiration monitoring sensor based on a piezoelectric-transducer-gated thin-film transistor (PTGTFT) aiming to measure respiration-induced dynamic force in real time with high sensitivity and robustness. It differs from passive piezoelectric sensors in that the piezoelectric transducer signal is rectified and amplified by the PTGTFT. Thus, a detailed and easy-to-analyze respiration rhythm waveform can be collected with a sufficient time resolution. The respiration rate, three phases of respiration cycle, as well as phase patterns can be further extracted for prognosis and caution of potential apnea and other respiratory abnormalities, making the PTGTFT a great promise for application in long-term real-time respiration monitoring.

## 1. Introduction

Respiratory diseases are the second leading (~6.5%) cause of mortality globally [1]. Fortunately, they are preventable if a reliable and accurate monitoring method is implemented. Therefore, real-time monitoring of respiratory rhythm that provides ample information about breathing trend becomes critical [2,3,4,5]. Moreover, study of respiratory patterns and phases helps diagnose pulmonary illness, such as apnea [6], asthma [7], and chronic obstructive pulmonary diseases [8]. Conventionally, electrocardiogram (ECG) [9,10] and transthoracic impedance plethysmography (TI-PPG) [11,12,13] are widely used to perform non-invasive monitoring of respiration rate. ECG monitors electrical stimulus response of lungs from multiple leads and TI-PPG monitors small change in tissue volumes upon respiration. Both require the examined body to be immobile during examination for accurate and reliable measurements. Therefore, such commercially-available devices are not well-suited for wearable and real-time monitoring in daily activities. In addition, they are bulky and consume a lot of power, which is inconvenient for long-term operation.

Other than ECG and PPG, various new wearable respiration rate sensors have also been investigated. Supplementary Figure S1 summarizes these sensors in terms of sensing mechanisms and sensing materials. Regarding sensing mechanisms, transduction of mechanical, electrical, thermal, and vaporous stimuli to electrical signals are typical. Among them, direct detection of air-movement upon respiration process using new-fashioned sensing materials has become popular since it is not affected by motion artifacts [14,15,16]. For example, a paper-based humidity sensor, adjustable inside a wearing mask, has been used for respiration rate monitoring through humidity measurement of conductive paper [14]. Impedance variation with water absorption of silica nanoparticles thin-film on flexible substrate was measured to extract respiration rate [16]. However, such sensors are very difficult to wear and are also affected by environmental humidity, thus giving unreliable data. Similarly, flexible and wearable pressure sensors can directly measure pressure change during the respiration process and they exhibit good features, such as being light and portable [17,18,19]. These sensors can be based on piezoelectric effect [20,21], capacitance change [22,23], and thermoelectric effect [24,25] in response of pressure change. However, they are passive devices with limited sensitivity and dynamic range. More recently, a hollow micro-structured pressure sensor offering a wide dynamic range has been reported [26]. However, it is non-contact based, limited to static pressure measurements, and its response time is problematic due to deformation dependence on ethylene-vinyl acetate (EVA) film structure. Respiration rate sensors based on triboelectric and pyroelectric effect with high sensitivity were very attractive [24,27]. However, their super high sensitivities lead to reliability issues arising from mechanical, motion, and electrostatic shocking. The long-term usage can also cause material/structural fatigue as a result of frequent friction.

In this work, we propose an active self-driven wearable piezoelectric sensor intended for real-time respiration monitoring. The device architecture will be presented along with the analytical modeling of sensitivity. The respiration rates and rhythm at multiple body peripheral during various physical activities will be obtained and the transient response of the sensor by detecting all respiratory patterns and phases will be also given. The feasibility of using this sensor for real-time respiration monitoring has been discussed particularly for mobile health care. Compared with other wearable respiration sensors, this proposed sensor is very competitive in terms of sensitivity, signal-to-noise ratio (SNR), dynamic range, operating voltage, power consumption, response time, and device fabrication process as listed in Table 1.

## 2. Methods

### 2.1. Sensor Architecture

**Figure 1:**
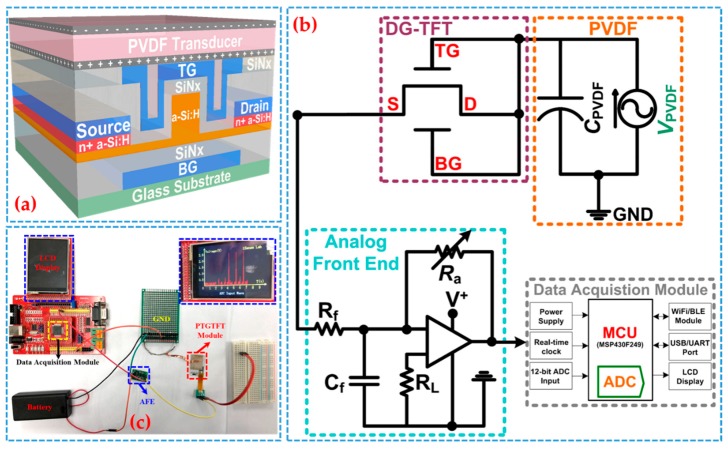
(**a**) Cross-sectional schematic illustration of a piezoelectric-transducer-gated thin-film transistor (PTGTFT) where a PVDF transducer is connected to a FIN-shaped a-Si:H piezoelectric transducer and a dual-gate thin-film transistor (DG-TFT); (**b**) equivalent circuit diagram of respiration monitoring sensor system composed of a PTGTFT, low-power analog front end (AFE) and conventional data acquisition module (MSP430F149); and (**c**) photo of the experimental setup for measuring the respiration rhythm signal by the proposed sensor system.

The cross-sectional schematic diagram of the proposed sensor is illustrated in Figure 1a. Different from a passive piezoelectric transducer with simply a sandwich structure, an active sensor is architected by a piezoelectric transducer and a dual-gate thin-film transistor (DG-TFT) forming a piezoelectric-transducer-gated TFT (PTGTFT). The sensor is capable of not only detecting a dynamic force signal but also rectifying it into a direct current (DC) signal and further amplifying it. The equivalent circuit diagram for respiration monitoring system based on the PTGTFT is shown in Figure 1b, where shortening bottom gate (BG), top gate (TG), and drain (D) terminals of the PTGTFT makes a diode-mode connection for signal rectification. Here, the DG-TFT is used instead of single-gate TFT for the sensitivity enhancement and better interfacing with the readout electronics. In this architecture, no external power source is needed and all the biasing voltages for the sensor originate from the PVDF transducer, making it self-driven with zero power consumption. The output DC current signal is further handled by an AFE consisting of a low-noise current amplifier and low-pass RC filter before entering the data acquisition module (DAM), which includes an ultra-low power microcontroller unit (MCU) that houses a built-in 12-digit analog-to-digital converter (ADC), an I/O interface, and a LCD panel. Figure 1c shows a photo of the proposed respiration monitoring sensor system.

### 2.2. Analytical Modeling

In the self-driven mode, the top gate voltage (*V_TG_*) is equal to the drain-source voltage (*V_DS_*) and the bottom gate voltage (*V_BG_*), and is given by the piezoelectric-induced charges (*Q_PVDF_*) over the total capacitance, *C_Total_*:

(1) $$V_{TG}=\;V_{BG}=\;V_{DS}=\;\frac{Q_{PVDF}}{C_{Total}}=\;\frac{Q_{PVDF}}{\left(C_{PVDF}+\;C_{TG}+\;C_{BG}\right)}$$

where *C_PVDF_*: Capacitance of the PVDF transducer, *C_TG_*: Top gate capacitance and *C_BG_*: Bottom gate capacitance in the DG-TFT. Threshold voltage (*V_T_*) of the DG-TFT is dependent with *V_TG_* as follows:

(2) $$V_{T}=\;V_{T_{O}}-\;\gamma .V_{TG}$$

where *V_To_* is the original threshold voltage of the DG-TFT with applying zero top-gate bias and γ indicates the variation in the *V_T_* with the *V_TG_*. The working regime of the PTGTFT depends on its force stimuli. In our previous studies [28,29,30], a similar device working in the deep-subthreshold regime is used to detect very weak dynamic forces from the heart beating for heart rate monitoring. The respiratory force is generally strong and thus the PVDF transducer generates voltages greater than *V_T_* (normally smaller than 1 V) while *V_DS_* > *V_BG_* − *V_T_* is readily satisfied. As a result, the PTGTFT likely works in the saturation region, giving the output current (*I_DS_*) as below:

(3) $$I_{DS}=\;\frac{1}{2}{µ}_{FE}\cdot C_{BG}\cdot \frac{W}{L}\cdot {\left[(V_{BG}-V_{T})\right]}^{2}$$

where µ*_FE_*: Field-effect mobility of the PTGTFT; W: Channel width of the PTGTFT, and L: Channel length of the PTGTFT. Substituting Equations (1) and (2) in Equation (3) gives:

(4) $$\sqrt{I_{DS}}=\;\sqrt{\frac{1}{2}{µ}_{FE}.C_{BG}.\frac{W}{L}}.\left[\left(\frac{Q_{PVDF}}{\left(C_{PVDF}+\;C_{TG}+\;C_{BG}\right)}\right)\left(1+\;\gamma \right)-\;V_{T_{O}}\right]$$

The sensitivity, *S*, in the saturation region can be defined as the ratio of the change of the square root of output current, $d\sqrt{I_{DS}}$, to the change of the generated charges by the respiration action, *dQ_PVDF_*, as follows:

(5) $$S=\;\frac{d\sqrt{I_{DS}}}{dQ_{PVDF}}=\sqrt{\frac{1}{2}{µ}_{FE}.C_{BG}.\frac{W}{L}}.\left[\left(\frac{\left(1+\;\gamma \right)}{\left(C_{PVDF}+\;C_{TG}+\;C_{BG}\right)}\right)\right]$$

As can be seen, the sensitivity is attributed to the material, design, and process parameters of µ_FE_, channel width and length (W/L) ratio, *γ*, *C_Total_*, and *C_BG_*, among which *γ* is a key parameter dictating how the transducer interacts and couples with the TFT. Our previous study has already found that the value of *γ* can be doubled by using a 3-D FIN-shaped TFT structure as opposed to a regular planar structure [30]. Hence, in this research, we designed and fabricated the PTGTFT with a 3-D FIN-shaped structure.

### 2.3. Sensor Fabrication

The fabrication process included preparation of the 3-D FIN-shaped DG-TFT based on amorphous silicon (a-Si:H) thin-film semiconductor material and processes [30] and the PVDF transducer that was formed by a sandwich structure of silver coated PVDF film (thickness: 52 µm; supplier: Measurement Specialties Inc., Hampton, VA, USA), i.e., Ag/PVDF/Ag. The process flow of fabricating 3-D FIN-shaped DG-TFT consisted of six photolithographic masks and was based on top–down approach of mainstream a-Si:H TFT process as shown in Supplementary Figure S2. Mask #1: A Chrome (Cr) metal layer of 100-nm thickness was deposited on glass substrate (Corning 1737) by direct current (DC) sputtering at room temperature followed by wet-etching to pattern photolithography of bottom gate. Mask #2: A 300-nm-thick SiN_X_ film as a bottom gate dielectric layer film and a 600-nm-thick a-Si:H film as an active semiconducting layer were deposited consecutively by a plasma-enhanced chemical vapor deposition (PECVD) at 300 °C in a single vacuum process. The second lithography step was patterned, followed by a selective dry etching process using inductively coupled plasma (ICP) reactive ion etcher at 10 °C to form the 3-D FIN-shaped structure. Mask #3: Ohmic contacts are formed by deposition of an additional layer of heavily doped n^+^-a Si:H of 50-nm thickness by PECVD at 300 °C. The third lithography was used to define TFT island followed by dry-etching of dual layers of n^+^-a-Si:H and a-Si:H. Mask #4: A 100-nm thick layer of Cr was deposited by DC sputtering and was then patterned by lithography to form source/drain electrodes followed by wet-etching processes. Afterwards, the n^+^-a-Si:H layer between the source/drain electrodes was removed using dry etching process. In the next step, 300-nm-thick SiN_X_ film was deposited as a top-gate dielectric layer by PECVD at 300 °C. Mask #5: The dielectric layer deposition is followed by the deposition of 100 nm Cr layer by DC sputtering and patterning by lithography and wet etching processes as top-gate electrode. Later on, the device was passivated with deposition 300-nm-thick SiN_X_ film by PECVD at 300 °C. Mask #6: In the final step, the contact vias were patterned by lithography and opened by dry-etching process for electrical probing. After the DG-TFT was prepared, the PVDF transducer was mechanically laminated over the DG-TFT on a glass substrate via low-temperature anisotropic conductive film (ACF) from Teamchem Materials Company. The laminator (Model: TWB-150; Company: Guangzhou KeFu Instrument Co., Guangzhou, China) was used to laminate PVDF over ACF, keeping the pressure at more than 0.3 MPa to completely crush the particles of interfacing ACF layer, and the temperature below 75 °C to retain the electrical properties of PVDF film (Supplementary Figure S3).

## 3. Results and Discussion

**Figure 2:**
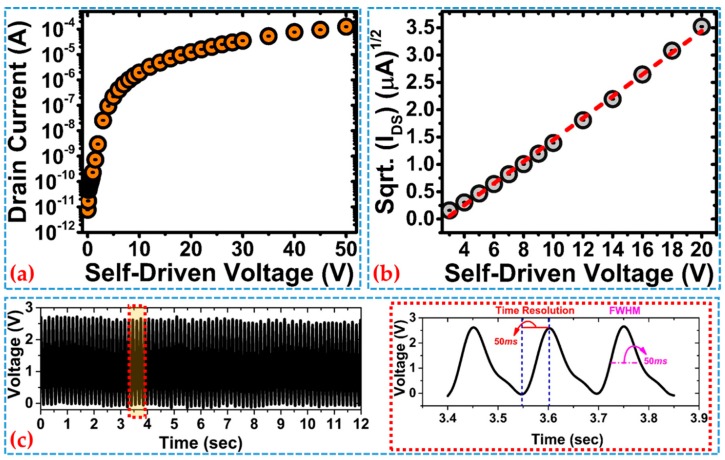
(**a**) Transfer characteristics of 3-D FIN-shaped DG-TFT, when operating in a self-driven mode; (**b**) Current variation of the fabricated 3-D FIN-shaped DG-TFT when operating in saturation region; (**c**) mechanical stability assessment and time-resolution evaluation of PTGTFT sensor at 6.5 Hz and force of 1 N.

Figure 2a shows the IV characteristics of a-Si:H dual-gate TFT with 3-D FIN-shaped channel structure in a self-driven mode, where *V_TG_* = *V_BG_* = *V_DS_*. The force generated from mechanical shock or sudden impact lies in the range of more than 10 N, which may produce output voltage peaks with tens of vs. [19,24]. For a highly-sensitive force sensor, a high voltage peak can be generated in a short time and may damage the AFE and cause reliability issue. However, in the case of the PTGTFT, the output signal upon mechanical or electrostatic shocking is high current instead of high voltage. From Figure 2a, we can observe that under self-driven voltage of 50 V, quite high voltage value to mimic the peak voltage received by the device, the output current is around 100 µA which can be safely handled by the AFE. In other words, the sensor is capable of bearing high voltages, thereby proving to have wide dynamic range and good reliability. Figure 2b illustrates that the square root of the output current varies with the self-driven voltage in a linear manner in accordance with Equation (4). The self-driven voltage generated from the PVDF transducer from the respiration process is typically around several voltages well above the threshold voltage of the DG-TFT. Figure 2c plots dynamic response of the sensor upon force load of 1 N at 6.5 Hz. The sensor is able to trace a dynamically-applied force with a rise time of 50 ms, sufficient for respiration monitoring.

**Figure 3:**
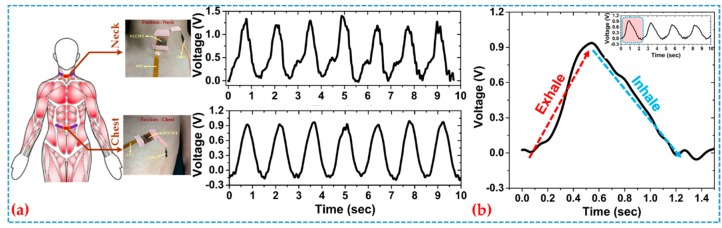
Dynamic response of the sensor at two peripheral points of (**a**) neck and chest; (**b**) signal of one complete respiration cycle.

Figure 3a shows the output voltage signals obtained from the AFE of the sensor system. The measurements were performed at the neck and the chest locations when a subject was at rest. Respiration rate was extracted from either the time difference between two peaks or the number of peaks over a specified time interval. The sensor was also collecting respiratory rhythms from multiple locations across the body. Figure 3b demonstrates that the signal increased and decreased as the subject exhaled and inhaled, respectively. In the chest location, the rib cage expanded during exhalation, making the PTGTFT experience an elevated force and leading to a signal increase. Contrarily, inhalation contracted the rib cage, driving the PTGTFT to experience a decreased force, thus resulting in the decrease of the output voltage.

**Figure 4:**
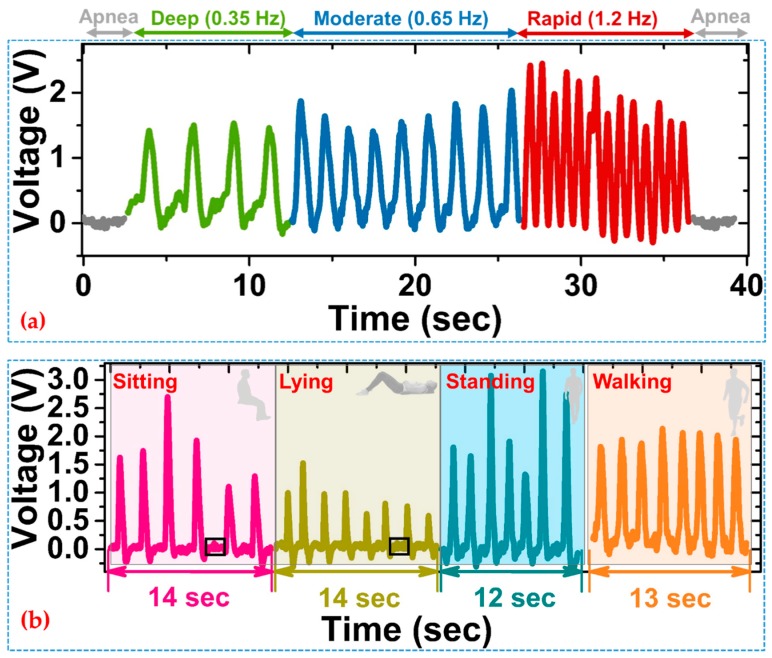
(**a**) Dynamic response of the sensor in different respiration modes at rest; (**b**) respiration monitoring tests for three different daily activities when the subject is (I) sitting; (II) lying; (III) standing, and (IV) walking.

Moreover, Figure 4a illustrates the output voltage signal of a monitored subject for different respiration modes, i.e., apnea, deep, moderate, and rapid. The time dependence of the voltage signals indicates the steady variation in the process of three breathing patterns. Shown in Figure 4a, as well, the zero voltage signal before and after different respiration modes corresponded to the subject holding the breath, thus showing the potential of indication for the diagnosis and warning of apnea. In addition to this, to demonstrate the sensor’s ability, the respiration rate of a subject was measured for an extended period of time (over one week), for two different circumstances of normal breathing and moderate breathing. The respiration rate was calculated and averaged for each position and was compared with the respiration derived from the ECG measurement (EDR). The resulted average value from both devices for each day is listed in Supplementary Table S1. The obtained results found no significant difference between PTGTFT and EDR measurements and infers the viability of PTGTFT for monitoring and analysis of respiratory activities of multi-subjects.

Furthermore, a series of experiments were conducted for the subject in various daily activities, to validate the portability of the sensor for real-time respiration monitoring. The respiration was monitored in four different states, i.e., sitting (state I), lying (state II), standing (state III), and walking (state IV). The experiment for state II was designed to evaluate and simulate the breathing process during sleep, while the other cases were designed to evaluate the respiration patterns of the subject in typical daytime activities. Figure 4b illustrates the signal variation during different breathing processes of sitting, sleeping, standing, and walking, respectively. The portion of the waveform where no signal was detected, e.g., in state I and state II, shows a clear sign of pause in a breath rhythm (black box). The measured respiration rates for the four states were 21/min, 26/min, 32/min, and 38/min, which are reasonable respiration rates for the healthy adults [27]. In the case of blank experiment or during breath holding (apnea), no signal occurred, which means that the generated signal corresponds to the physiological parameter. It also indicated that mechanical motion of monitored individual can be acquired by the PTGTFT. Therefore, in the event of coughing, where the air is entrapped in the lungs due to shutting of vocal cords, the rib-cage muscle experiences the substantial force, resulting in the sudden rise of output voltage as shown in Supplementary Figure S5, thus showing the effectiveness of the PTGTFT towards additional monitoring of human motion and activities, e.g., swallowing, sneezing, etc.

**Figure 5:**
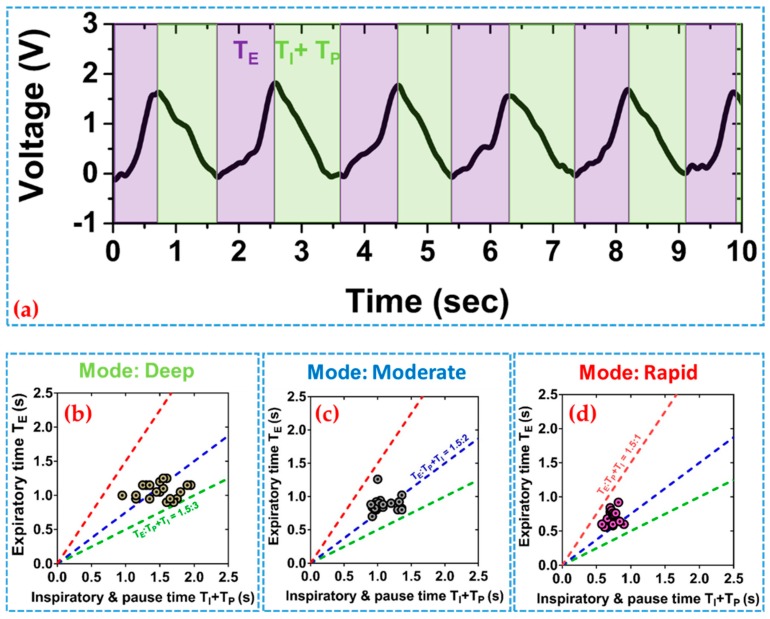
(**a**) Phase analysis of human respiration rhythm; relation of expiratory time and sum of inspiratory and pause time in during respiration mode of (**b**) deep, (**c**) moderate, and (**d**) rapid.

To evaluate normality of human breathing patterns, three phases of respiration are examined: Inspiratory, expiratory, and pause phase. Thanks to the quick dynamic response this sensor has, phases of respiration can be accurately monitored. Figure 5a shows a typical voltage waveform obtained from a series of respiration cycles where the three respiration phases in each respiration cycle are highlighted. The expiratory time (T_E_) corresponds to the time of signal rise and the inspiratory time (T_I_) and pause time (T_P_) correspond to the time when the signal decreases and the signal keeps at the minimum. Figure 5b–d presents the relation of T_E_ and T_I_ + T_P_ for each respiration mode of deep, moderate, and rapid breathing for a monitored subject, and the patterns distribute around the lines of T_I_ + T_P_:T_E_ = 1:1.5, 2:1.52, and 3:1.5, respectively. When inhaling and exhaling are successive without being stopped, it means T_P_ is much smaller than T_E_ and T_I_, and the plot moves to the red line of 1:1.5. Similarly, when the subject is experiencing deep breathing, the pause time will increase and the plot will move towards the green line of 3:1.5, which is equal to the usual I:E ratio [6]. Thus, phase analysis of respiration patterns can give a better understanding of human respiratory dynamics. Our study shows that the proposed sensor enables efficient collection of respiration signal waveforms for real-time monitoring that could be utilized during various medical assessments similar to the application of a fiber-optic sensor to study the relationship between anxiety and respiratory rates for the patients undergoing MRI treatment [31]. In such application scenarios, the PTGTFT would be potentially capable of acquiring and analyzing respiratory activities during intermittent stages of assessment as well.

## 4. Conclusions

An active self-driven sensor for real-time respiration monitoring was proposed and studied. An analytical model was established to elaborate its sensitivity. Such an active sensor differs from passive sensors in that it provides signal rectification and amplification through a piezoelectric-transducer-gated TFT. The highly-sensitive and reliable sensor allows for monitoring respiratory activities at different body locations and analysis of respiration phases in real time.

## Figures and Tables

**Table 1:** Summary of this research compared with the prior arts in terms of sensitivity, signal-to-noise ratio (SNR), dynamic range, driving voltage, response time, and power consumption.

Sensor System	Operating Voltage (V)	Power Consumption (W)	Response Time (sec)	Dynamic Range	Sensitivity (@ 0.5N)	SNR (dB)	Device Process
Triboelectric Based Sensing Method [27]	Self-Driven (~0 V)	<1 m	~100 m	~ 0.2 N to 10 N	~600 mV	45	Low cost and simple
Humidity Based Measurement [16]	>5 V	~20 m	~700 m	——	Low	——	Low cost and complex
Optical Based Sensing Method [12,13]	>3 V	~60 m	>1000 m	——	Low	Low	Medium cost and complex
***This Work***	**Self-Driven (~0 V)**	**~600 µ**	**50 m**	**> 50 mN**	**~800 mV**	**>20**	**Low cost and simple**

## Supplementary Materials

The following are available online at https://www.mdpi.com/1424-8220/19/14/3241/s1, Figure S1: Summary of recently-developed wearable respiration rate sensors, Figure S2: Schematic illustration of fabrication steps of DG-TFT, Figure S3: schematic illustration of PTGTFT fabrication by the integration of PVDF film and 3-D FIN-shaped DG-TFT, Figure S4: Electrical characterization of 3-D FIN-shaped DG-TFT, Figure S5: Electric response of the PTGTFT to successive mechanical motions; Table S1: Comparison of respiration rate monitoring between PTGTFT and EDR.
