# Supplementary material for: An Active Self-Driven Piezoelectric Sensor Enabling Real-Time Respiration Monitoring
Source: Sensors (Basel). 2019 Jul 23;19(14):3241. doi: 10.3390/s19143241 (PMC6679499; doi:10.3390/s19143241)
Supplement: Supplementary file 1 [file sensors-19-03241-s001.pdf]

Supplementary Information

for

An Active Self-Driven Piezoelectric Sensor Enabling Real-Time Respiration Monitoring

Ahmed Rasheed [1,2], Emad Iranmanesh [1], Weiwei Li [1,2], Yangbing Xu [1,2], Qi Zhou [1,2], Hai Ou [1] and Kai Wang [1,2]*

[1] Guangdong Province Key Laboratory of Display Material and Technology, State Key Laboratory of Optoelectronic Materials and Technologies, School of Electronics and Information Technology, Sun Yat-sen University, No. 132 East Waihuan Road, Guangzhou 510006, P. R. China.

[2] Sun Yat-sen University Shunde Research Institute, No. 9 Eastern Nanguo Road, Shunde District, Foshan 523800, P. R. China.

* Correspondence: wangkai23@mail.sysu.edu.cn

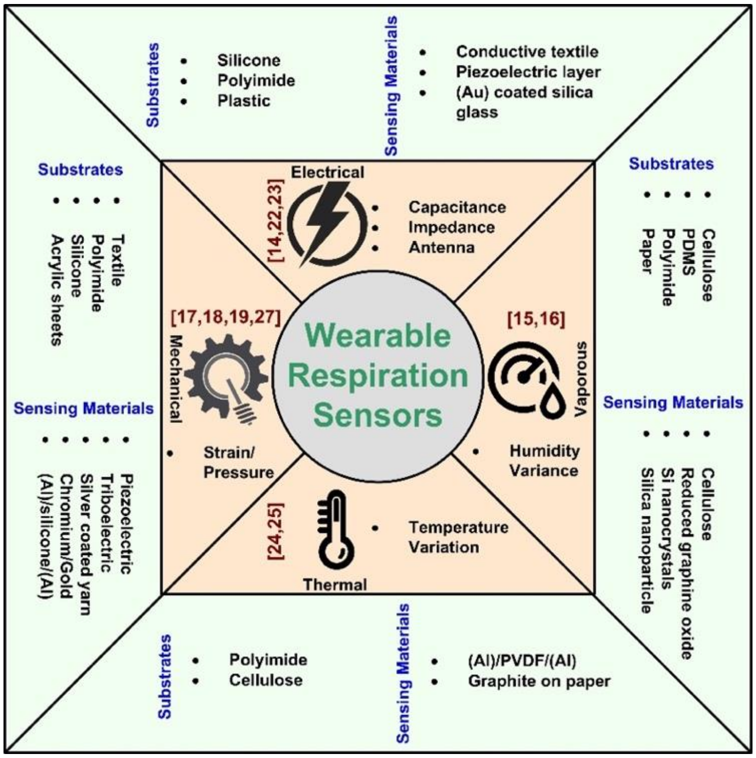

Supplementary Figure 1. Summary of recently-developed wearable respiration rate sensors. The main categories of signal transduction are based on electrical, mechanical, thermal and humidity stimuli.

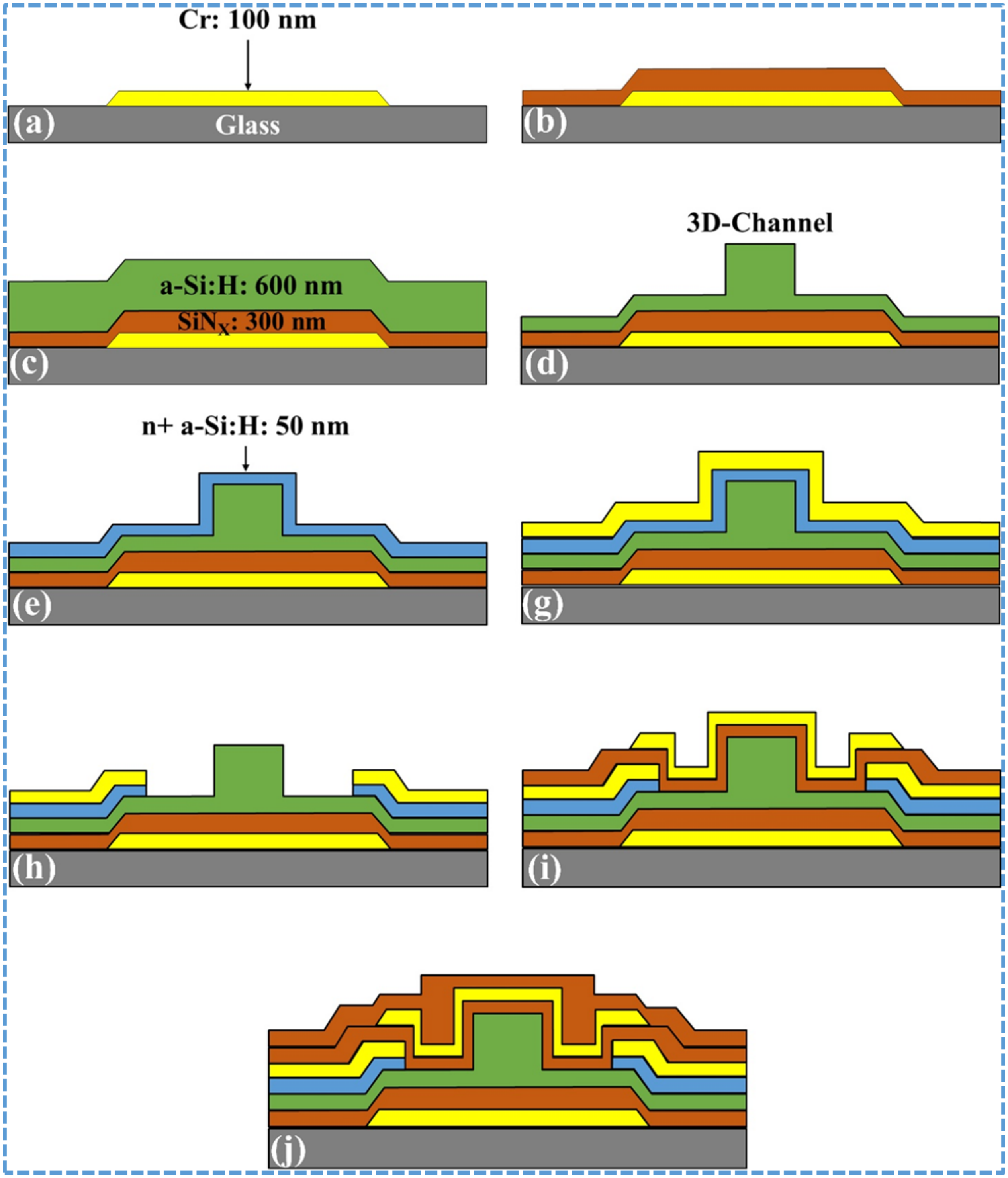

Supplementary Figure 2. Device layouts and fabrication steps of 3-D FIN-shaped a-Si:H dual-gate thin film transistor (DG-TFT) on a glass substrate. Schematic illustration of fabrication steps of DG-TFT.

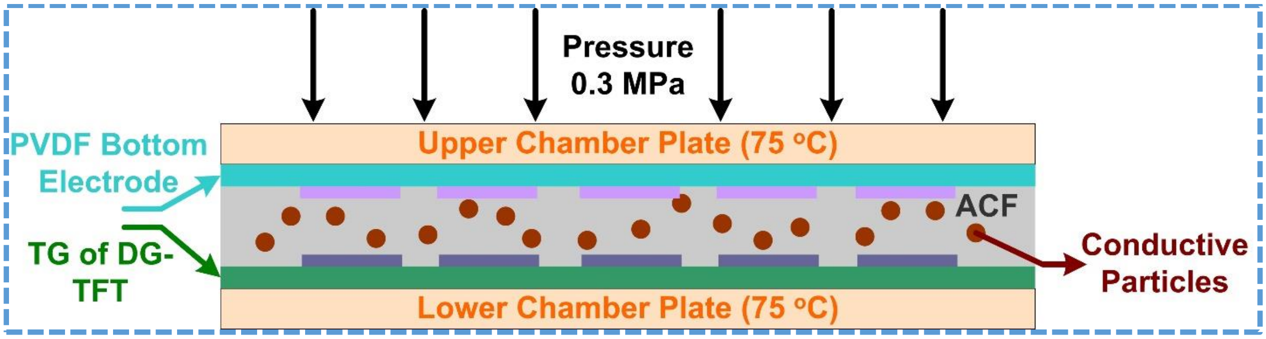

Supplementary Figure 3. Device layout and integration process of piezoelectric transducer film and 3-D FIN-shaped a-Si:H dual-gate thin film transistor (DG-TFT) on a glass substrate. Cross-sectional schematic illustration of PTGTFT fabrication by the integration of PVDF film and 3-D FIN-shaped DG-TFT.

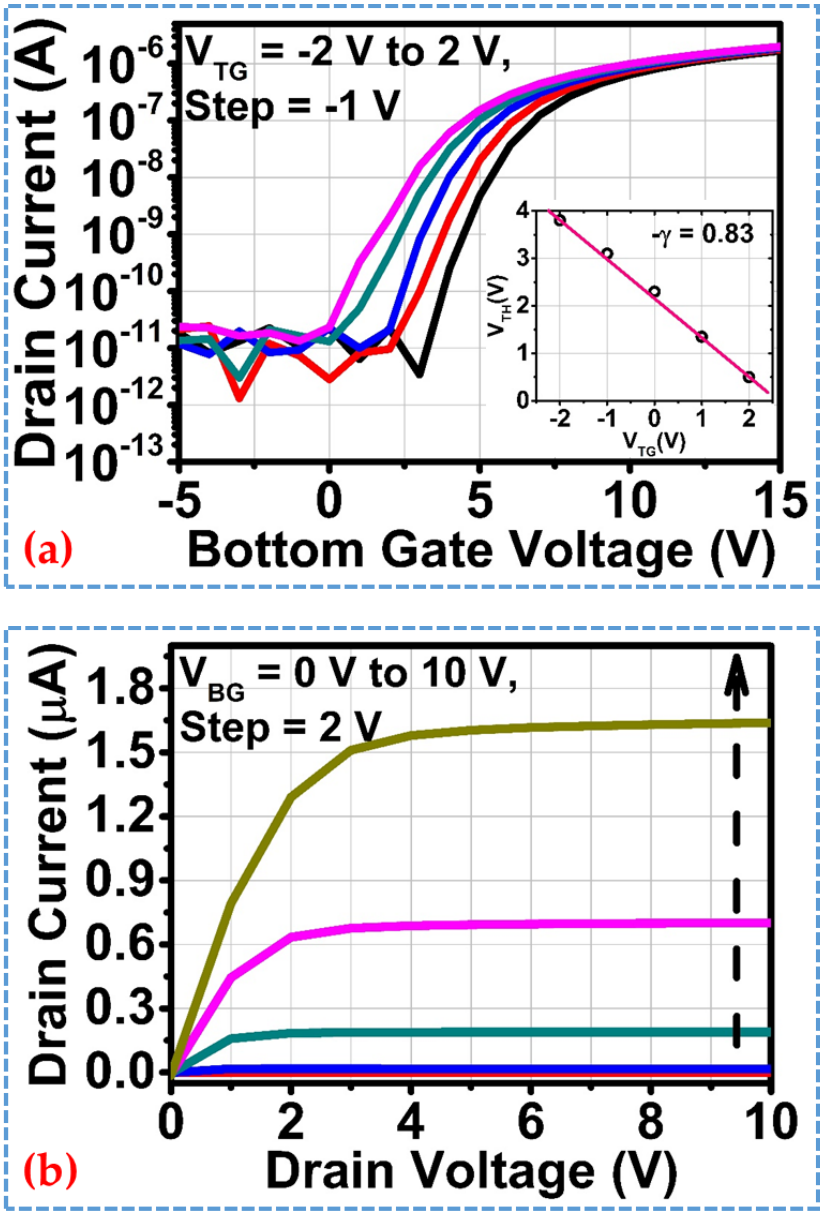

Supplementary Figure 4. Electrical characterization of 3-D FIN-shaped a-Si:H dual-gate thin film transistor (DG-TFT) on a glass substrate. (a) Transfer characteristics, inset shows value of the extracted dependence parameter (γ) and **(b)** Output characteristics.

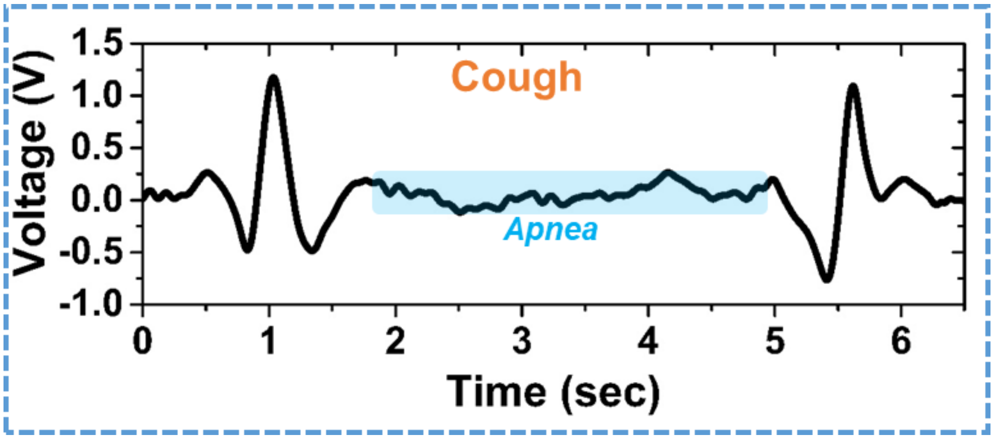

Supplementary Figure 5. Electric response of the PTGTFT to successive mechanical motions (cough and holding breath).

Supplementary Table 1. The averaged respiration rate of a single subject over multiple days for normal and moderate breathing acquired from the PTGTFT at two peripheral points (neck and chest) and ECG-derived-respiration (EDR).

**Measurement Day #**	**Normal Breathing**			**Moderate Breathing**		
	**Neck Position**	**Chest Position**	**EDR**	**Neck Position**	**Chest Position**	**EDR**
**1**	17 ± 2.6	17.5 ± 2.5	18	21 ± 2.5	20.5 ± 1.5	22
**2**	14.6 ± 1.06	14 ± 1.5	15.5	19 ± 1.1	20.7 ± 1.3	20
**3**	16.5 ± 2.3	16.5 ± 1.6	16.5	19 ± 0.5	19 ± 0.9	20
**4**	17.3 ± 0.9	17 ± 1.2	18.5	22 ± 2.3	21 ± 1.5	19.5
**5**	14.5 ± 2.1	15 ± 1.1	15.5	18 ± 1.4	17 ± 2.1	17.5
**6**	16 ± 2.2	16 ± 1.5	17	20 ± 0.9	20 ± 1.1	20
